# Photoinactivation of *Staphylococcus aureus* using protoporphyrin IX: the role of haem-regulated transporter HrtA

**DOI:** 10.1007/s00253-015-7145-5

**Published:** 2015-12-03

**Authors:** Joanna Nakonieczna, Monika Kossakowska-Zwierucho, Michalina Filipiak, Weronika Hewelt-Belka, Mariusz Grinholc, Krzysztof Piotr Bielawski

**Affiliations:** Intercollegiate Faculty of Biotechnology, University of Gdansk and Medical University of Gdansk, Kladki 24, 80-822 Gdansk, Poland; Faculty of Biology, University of Gdansk, Wita Stwosza 59, 80-308 Gdansk, Poland; Faculty of Chemistry, Gdansk University of Technology, Gabriela Narutowicza 11/12, Gdansk, Poland

**Keywords:** Protoporphyrin IX, Photoinactivation, Membrane fluidity, Lipid content, *Staphylococcus aureus*

## Abstract

Light- and photosensitiser-based antimicrobial photodynamic therapy is a very promising approach to the control of microbial infections. How the phenotypic features of a microorganism affect its response to photosensitiser-based photokilling represents an area of substantial research interest. To understand the mechanisms governing the phenomenon of a strain-dependent response to photodynamic inactivation (PDI), we analysed the possible role of the membrane-located haem transporter HrtA in *Staphylococcus aureus*. We used a *S. aureus* strains with an inactivated component of the haem-regulated transporter, HrtA, along with its wild-type counterpart to determine differences in PDI outcome and photosensitiser uptake between the studied isogenic strains. We observed that a lack of HrtA protein potentiates the phototoxic effect towards *S. aureus* but only when extracellular protoporphyrin IX is used. The observed effect may depend on the function of the HrtA transporter but is likely to result from changed membrane properties following the absence of the protein in the membrane. This indicates that disturbing the membrane properties is an attractive method for improving the efficacy of the photodynamic inactivation of microorganisms.

**Electronic supplementary material:**

The online version of this article (doi:10.1007/s00253-015-7145-5) contains supplementary material, which is available to authorized users.

## Introduction

Photodynamic inactivation of bacterial pathogens is becoming an interesting therapeutic option to treat microbial infections; however, this method can also be utilised in food decontamination (Tortik et al. 2014) and environmental applications (Almeida et al. 2014). Previously, the method has primarily been used in cancer treatments, especially in countries in which it was clinically approved in the late 1980s (Prout et al. 1987). Soon thereafter, both Gram-positive and Gram-negative bacteria were found to be killed using this method, and antimicrobial photodynamic chemotherapy was developed (Merchat et al. 1996; Wilson et al. 1995). The related photodynamic action is caused by three elements, namely light, a photosensitiser and oxygen. A photosensitiser, which is usually a small molecular compound, accumulates in microbial cells and, upon illumination with light of an appropriate wavelength, becomes excited into its triplet state. The energy produced during excitation is transmitted from a photosensitiser via electron transfer to either (1) cellular substrate and then to oxygen to form several reactive oxygen species (type I mechanism) or (2) transferred directly to molecular oxygen to form a very reactive singlet oxygen (type II mechanism), which is primarily responsible for phototoxic damage in target cells. Various biomolecules are affected by the photodynamic action, namely proteins (e.g. aromatic amino acids), nucleic acids and unsaturated lipids, depending on the nature of the photosensitiser (charge and polarity) and its subcellular localisation (Ochsner 1997). Effective photosensitisers, i.e. those that cause a significant (at least 4-log) decrease in cell survival, are believed to localise in the cell membrane and express cytotoxic effects towards membrane components (Ooi et al. 2009; Oriel and Nitzan 2012). This is the mechanism by which porphyrin-based photosensitisers are believed to act (Ooi et al. 2009). The effectiveness of photodynamic inactivation (PDI) treatment is generally independent of the antimicrobial resistance pattern; thus, multiresistant bacteria, such as methicillin-resistant *S. aureus*, can be eradicated with photodynamic treatment with efficiency similar to its methicillin-sensitive counterpart (Maisch et al. 2007; Schastak et al. 2010). The singlet oxygen and other reactive oxygen species-based mechanisms (highly reactive free radicals) of photodynamic action assume that the development of resistance to such treatments is highly unlikely (Giuliani et al. 2010; Tavares et al. 2010). From a clinical point of view, the development of effective therapeutic treatments that do not induce resistance is of high interest, particularly in cases when the treatment must be performed several times.

Thus far, it has not been demonstrated that resistance to photodynamic inactivation can be induced even with low doses of light. However, we observed that clinical isolates of the same species present significantly different phenotypic variations in response to porphyrin-based photodynamic treatment. The observed differences ranged from a 0.2 log_10_ reduction of colony-forming unit (CFU) per millilitre, representing strains with elevated resistance to PDI, to a 5 log_10_ reduction of CFU per millilitre in vulnerable strains. Several attempts were undertaken to elucidate the mechanism responsible for this phenomenon; however, only biofilm production was shown to significantly affect the effectiveness of the photodynamic process (Grinholc et al. 2008). Biofilm production, however, is not the only factor contributing to these differences in survival upon photodynamic treatment; in the group of weak biofilm producers, *Staphylococcus aureus* strains with elevated resistance to PDI were also observed. Several other factors can potentially contribute to a particular response of the strain to photodynamic treatment. The activity of enzymes that detoxify reactive oxygen species, which arise as a consequence of PDI treatment, can be a contributing factor. However, superoxide dismutases, which inactivate superoxide anions, have been shown to have only secondary effects on PDI outcome (Nakonieczna et al. 2010).

Currently, little is known about the mechanism by which bacterial cells accumulate exogenous porphyrins. The haem transport system is suggested to play a crucial role in this process (Moriwaki et al. 2011). Different metalloporphyrins are recognised by a well-characterised staphylococcal iron-regulated surface determinant system (Isd), namely the IsdH haem receptor, and then transferred to IsdA. This indicates that the Isd system may be used by these antibacterial compounds to access the interior of the cells (Moriwaki et al. 2011). The IsdC protein was also demonstrated to predominantly bind to protoporphyrin IX (PpIX) and to haem to a lesser extent (Mack et al. 2004). Another system that is involved in haem import in *S. aureus* cells is the membrane-localised ATP-binding cassette (ABC) transporter HtsA (Skaar et al. 2004). however, this particular import system is more engaged in staphyloferrin A transport (Beasley et al. 2009). Notably, *S. aureus* is very sensitive to haem and possesses systems that can alleviate the toxicity of this compound. HrtA and HrtB are two cooperating proteins that protect bacterial cells from the negative effects of haem accumulation (Torres et al. 2007). Based on literature data, HrtAB is an ABC transporter, where HrtA acts as ATPase and HrtB is an integral membrane permease forming a substrate transport channel (Stauff et al. 2008). HrtA is upregulated in bacterial cells upon haem presence; however, currently, there are no published data on steady state level of HrtA protein in cells (Friedman et al. 2006). The process of HrtA production is regulated by haem sensor system HssRS, which activates HrtAB (Stauff et al. 2007).

Based on the published data indicating that haem transporters may have a role in porphyrin transport, we investigated the possible role of these transporters in the accumulation of protoporphyrin IX and the potential of photodynamic killing of *S. aureus*. We showed that *S. aureus* mutant lacking HrtA haem transport system accumulated the largest amount of PpIX and was the most vulnerable to photodynamic treatment. However, the observed phenomenon was not dependent on the function of the protein but rather on secondary effects related to its physical absence in the membrane. This indicates that membrane perturbations may improve the efficacy of photodynamic killing via PpIX and potentially other photosensitisers that interact with the bacterial membrane.

## Materials and methods

### Bacterial strains and culture media

This study was conducted with four *S. aureus* strains: (i) *S. aureus* Newman NCTC 8178 clinical isolate, a wild-type strain (Duthie and Lorenz 1952). (ii) the *S. aureus* ΔIsdD *isd::erm* strain, in which the gene responsible for haem import was replaced with a resistance cassette to erythromycin (Mazmanian et al. 2003). (iii) *S. aureus* ΔHtsA, which was obtained via allelic replacement (Mason and Skaar 2009). and (iv) *S. aureus* ΔHrtA, which was obtained via allelic replacement (Torres et al. 2007). All of the bacterial strains were provided by Dr. Eric P. Skaar from the Department of Microbiology and Immunology at the Vanderbilt University Medical Center. The bacteria were grown in tryptic soy broth (TSB) (Biomerieux, Marcy l’Etoile, France). To prepare divalent metal ion-free medium, TSB was treated with a Chelex^®^-100 chelating ion exchange resin for 6 h and supplemented with 400 μM MgSO_4_. When necessary, Chelex-treated TSB medium was supplemented with FeSO_4_ to a final concentration of 20 μM. The *S. aureus* ΔIsdD strain was cultured in the presence of 10 μg erythromycin ml^−1^ (Fluka, Buchs, Switzerland).

### Chemicals

PpIX was purchased from Sigma-Aldrich^™^; 1 mM solution was prepared in dimethyl sulfoxide (DMSO) and stored in the dark at room temperature. 5-Aminolevulinic acid hydrochloride (5-ALA) was purchased from Fluka, Switzerland; a 100 mM solution was prepared in phosphate-buffered saline (PBS), pH 6.5, and kept at 4 °C for at most 1 week. A new methylene blue (Sigma-Aldrich^™^, Munich, Germany) 10 mM solution was prepared in deionised water and stored at −20 °C. All solvents and other chemicals were of analytical grade, with the exception of those used for experiments with liquid chromatography–mass spectrometry (LC-MS), in which high-performance liquid chromatography (HPLC) and LC-MS grade solvents were used.

### Photosensitiser accumulation

*S. aureus* strains were grown overnight and adjusted to optical density (OD)_600_ = 0.3. Protoporphyrin IX was added to 800-μl bacterial aliquots to final concentrations in the range of 1–20 μM (for PpIX) and 30–300 μM (for new methylene blue (NMB)). Samples were incubated for 30 min at 20 °C in darkness. This temperature was used to assure optimal functioning of the HrtA protein (Stauff et al. 2008). After incubation, the bacterial cells were washed twice with PBS and bacterial cell lysates were prepared by incubating cells in a 0.1 M NaOH/1 % sodium dodecyl sulfate (SDS) (*w*/*v*) solution for 24 h at room temperature. The fluorescence intensity of 100 μl of each sample was measured spectrophotometrically with the use of a Victor^™^ Multilabel Plate Reader (PerkinElmer, Boston, MA, USA). The PpIX concentration was obtained from a calibration curve that was prepared based on a known concentration of PpIX in a 1 M NaOH/1 % SDS (*w*/*v*) solution. In the experiments with trypsin pre-treatment, cells were grown overnight and adjusted to OD_600_ = 0.3 and 800-μl bacterial aliquots were centrifuged (1 min, 7500×*g*) and further dissolved in the same volume of 0.005 % (*w*/*v*) concentration of the trypsin. Following incubation with trypsin for 15 min, 37 °C, cells were centrifuged (2 min, 7500×*g*) and washed twice with PBS. Samples were incubated with PpIX for 30 min at 37 °C in darkness and twice washed with PBS. The cells were then subjected to lysis and measurements as described above. Uptake values were presented as PpIX molecules accumulated per cell according to the following formula:

$$ \mathrm{PpIX}\;\mathrm{molecules}\;\mathrm{accumulated}\;\mathrm{per}\;\mathrm{cell}=\frac{\mathrm{PpIX}}{\mathrm{Mw}\;\mathrm{PpIX}}\times \raisebox{1ex}{$\mathrm{N}\mathrm{A}$}\!\left/ \!\raisebox{-1ex}{$\mathrm{C}\mathrm{F}\mathrm{U}$}\right., $$

where PpIX is the amount of molecules obtained from a calibration curve based on known concentrations of PpIX, Mw PpIX is the molecular weight of PpIX (562.6 g/mol), NA is the Avogadro’s number (6.022 × 10^23^), and CFU is the colony-forming unit obtained using serial dilutions counted for 1 ml of the analysed samples.

### Photoinactivation experiments

*S. aureus* strains were grown overnight and adjusted to OD_600_ = 0.055–0.06. A specific photosensitiser was added to 800-μl aliquots of each bacterial strain to a final concentration of 0–50 μM (PpIX) or 20 μM (NMB). Samples were incubated in the dark at 37 °C for 30 min. Then, 100 μl of each sample was transferred into a 96-well plate and illuminated with a specific dose of light. Bacterial survival after photoinactivation was estimated by plating serially diluted bacterial suspensions on tryptic soy agar (TSA) plates. CFUs were counted, and the survival fraction was calculated as a percentage of the surviving bacteria with respect to the surviving bacteria in the untreated sample and presented in decimal logarithmic scale. The results of each experiment are presented as the mean of at least three independent replicates with the standard deviation of the mean.

### Illumination method

Both red and blue light were used in photoinactivation experiments with PpIX. Red light was applied for illumination protocol with NMB as a photosensitiser. Blue light was used in the experiments with 5-ALA treatment. The light (blue light, 385–480 nm, and red incoherent polarised light, 620–780 nm) was generated using a Q.Light PDT lamp (Q Products AG, Rorschach, Switzerland) with changeable filters (www.qlight.info). The lamp delivers a power of 70 mW/cm^2^ (4.2 J/cm^2^/min) at a maximum treatment distance of 16.5 cm with a treatment diameter of 16.5 cm and depth of penetration of 3–4 mm. Bacteria were illuminated in a 96-well plate in aliquots of 100 μl. Light doses ranging from 0 to 500 J/cm^2^ were obtained using the red filter, whereas doses that ranged from 0 to 300 J/cm^2^ were obtained using the blue filter. During illumination, the temperature of bacterial suspensions did not exceed 37 °C. After illumination, 10 μl of bacteria treated with a particular dose of light were collected for serial dilutions and plating.

### Induction of porphyrin production with 5-ALA

Overnight cultures of *S. aureus* were refreshed in TSB medium to OD_600_ = 0.1. The cells were grown with aeration until they reached OD_600_ = 0.4. The cultures were divided into five batches of 15 ml each and centrifuged for 10 min at 4000 rpm at 8 °C. After washing with PBS, the bacteria were resuspended in 10 ml of PBS and 5-ALA was added to final concentrations of 0.01, 0.05, 0.5 and 1 mM. The bacteria were placed in a shaking incubator (100 rpm) at 37 °C for 4 h in the dark. Subsequently, 100-μl aliquots were collected and photoinactivation was performed according as previously described (photoinactivation experiments). In these experiments, blue light (50 J/cm^2^ light dose) was used. We chose the blue light in our experimental setup with ALA-PDI as originally this type of light was the most extensively studied and finally approved for treatment of acne in USA. Besides, the wavelength in the range of blue light covers the Soret band of PpIX absorption, which is the highest absorption peak within PpIX whole emission spectrum. Bacterial survival after photoinactivation was estimated by plating. The survival rate was presented using a decimal logarithmic scale according to each given concentration of 5-ALA. After incubation with 5-ALA, endogenously produced porphyrins were extracted from the bacteria using an NH_4_OH:acetone solution (1:9 *v*/*v*). The bacteria were centrifuged for 5 min at 4000 rpm at 4 °C, washed with PBS and resuspended in 300 μl of an NH_4_OH:acetone (1:9 *v*/*v*) solution. The samples were vigorously shaken for 2 min. Bacterial cell extracts were centrifuged for 5 min at 9000 rpm at room temperature. The fluorescence of the extracted porphyrins in the supernatants was measured using a Victor^™^ Multilabel Plate Reader (PerkinElmer, Boston, MA, USA). The concentration of intracellular porphyrins was estimated based on the calibration curve prepared from the known concentrations of protoporphyrin IX in a solution of NH_4_OH:acetone. The porphyrin amount was presented as the concentration (nM) per number of cells, which were estimated by plating. Three independent biological replicates were conducted.

### Fluorescence-activated cell sorting analysis

Fluorescence-activated cell sorting (FACS) was used to detect the increased production of endogenous porphyrins in living cells (Nitzan and Kauffman 1999). Bacteria incubated with 5-ALA, as described in the previous section, were collected in aliquots of 25 μl and washed with PBS containing 1 % BSA (*w*/*v*). Finally, 1-ml samples were analysed on a flow cytometer (Becton Dickinson, Franklin Lakes, NJ, USA). Fluorescence emission was excited with a 488-nm laser light. The fluorescence signal of 5-ALA-treated cells is presented with respect to non-ALA-treated samples. The percentage of fluorescent cells corresponds to the fraction of cells with increased porphyrins produced upon 5-ALA induction with respect to the control cells, which were not incubated with 5-ALA.

### Cell membrane fluidity assay

Temperature-dependent membrane fluidity was quantified by measuring the fluorescence anisotropy with a 1.6-diphenyl-1.3.5-hexatriane (DPH) probe (Sigma-Aldrich^™^, Munich, Germany), according to modified protocols described by Bayer and Voss (Bayer et al. 2000; Voss and Montville 2014). A 2-mM stock solution of DPH was prepared in tetrahydrofuran, and 4 μM working solution was prepared by adding 100 μl to 50 ml of 0.05 M Tris–HCl (pH 7.6). Residual tetrahydrofuran was removed via gentle flushing with nitrogen. The solution was stored in the dark at 4 °C until use. Whole-cell suspensions of each bacterial strain were prepared with a bacterial density of 4.5 McFarland units (10^8^ CFU/ml) in TSB medium. Suspensions were pelleted via centrifugation (5000×*g*, 15 min) and then resuspended in 500 μl of digestion buffer (20 % [*w*/*v*] sucrose, 0.05 Tris–HCl and 0.145 M NaCl [pH 7.6]). The bacterial cell wall was then digested with 0.8 U of lysostaphin (A&A Biotechnology, Gdynia, Poland) in the presence of 6 U of DNAse I (EURx, Gdansk, Poland) for 1 h at 37 °C. Protoplasts were collected via centrifugation (9000×*g*, 15 min) and resuspended in 200 μl of fresh digestion buffer. The adequacy of cell wall digestion was confirmed via Gram staining. For DPH labelling, protoplasts suspended in digestion buffer were mixed with DPH solution at a 1:1 ratio to obtain a 2 μM final concentration and incubated in the dark at 30 °C for 45 min. A JASCO Spectrofluorimeter FP-8500 (Japan) was used for fluorescence anisotropy measurements. The analysis was carried out with a 300 μl volume of a labelled cell suspension agitated at 200 rpm in a temperature gradient ranging from 20 to 40 °C (ramping rate 1 °C per 1 min). Above this temperature, a disruption of labelled protoplasts was observed using fluorescence microscopy. The measurement parameters included a vertically polarised excitation wavelength of 360 nm (bandwidth 5 nm) and emission wavelength of 426 nm (bandwidth 10 nm) through a rotating polariser. The signal was measured for 2 s at each 2.5 °C interval. Each experiment was performed in three independent biological replicates. Fluorescence anisotropy (*r*) was calculated according to the following equation:

$$ r=\frac{I_v-{I}_p}{I_v+2\kern0.5em {I}_p}, $$

where *I*_*v*_ and *I*_*p*_ represent the fluorescence intensity measured with parallel and perpendicular orientations of the analyser, respectively.

### Lipid fingerprinting using liquid chromatography and quadrupole time-of-flight mass spectrometry

Samples and bacterial growth conditions were used as previously described (Hewelt-Belka et al. 2014). Bacteria were cultured in 50-ml bovine heart infusion (BHI) medium (BioMerieux, Marcy l’Etoile, France) for 23 h in 37 °C. The cells were harvested via centrifugation (5 min, 7000×*g*, 20 °C), washed twice with 0.78 % NaCl and further lyophilised for 23 h. Of lyophilised cells, 15 mg was dissolved in 500 μl of deionised water, and extraction was performed in borosilicate glass tubes according to the modified Bligh and Dyer method (Bligh and Dyer 1959). Briefly, 1.9 ml of chloroform:methanol mixture (1:2 *v*/*v*) and 600 mg of glass beads (0.10–0.11 mm diameter; Sartorius, Goettingen, Germany) were added to bacterial cells. The mixture was then vortexed (5 min). Next, 625 μl of chloroform was introduced, followed by 10 s vortexing and the addition of 625 μl of deionised water and another 60 s of vortexing. Afterwards, the sample was centrifuged at 5000 × g for 10 min to remove the delipidated cells that remained. The lower organic phase containing lipids was gently aspirated by a glass Pasteur pipette and transferred to a clean glass tube and subsequently analysed via liquid chromatography and quadrupole time-of-flight mass spectrometry (LC-Q-TOF-MS). All of the chemicals used were of HPLC and MS grade. Six *S. aureus* HrtA and six *S. aureus* Newman culture samples were prepared and processed with the same analytical procedure. Samples were analysed on an Agilent 1290 LC system coupled to a 6540 Q-TOF-MS with a dual ESI source (Agilent Technologies, Santa Clara, CA, USA). The data analysis was performed using MassHunter Workstation Software Qualitative Analysis, version B.03.01 (Agilent Technologies, Santa Clara, CA, USA) and Microsoft Excel 2010 (Microsoft, Redmond, USA) according to a previously described method (Hewelt-Belka et al. 2014).

### Statistical analysis

Each experiment was performed at least in triplicate. The primary data are presented as the means with standard deviations of the mean. The statistical analysis was performed using a one-way analysis of variance (ANOVA) with Tukey’s post hoc test. The hypotheses were tested at a significance level of 0.05. All analyses were performed using the STATISTICA version 10.0 software (StatSoft Inc. 2011, data analysis software system, Tulsa, OK, USA).

## Results

### PDI survival assays

**Fig. 1:**
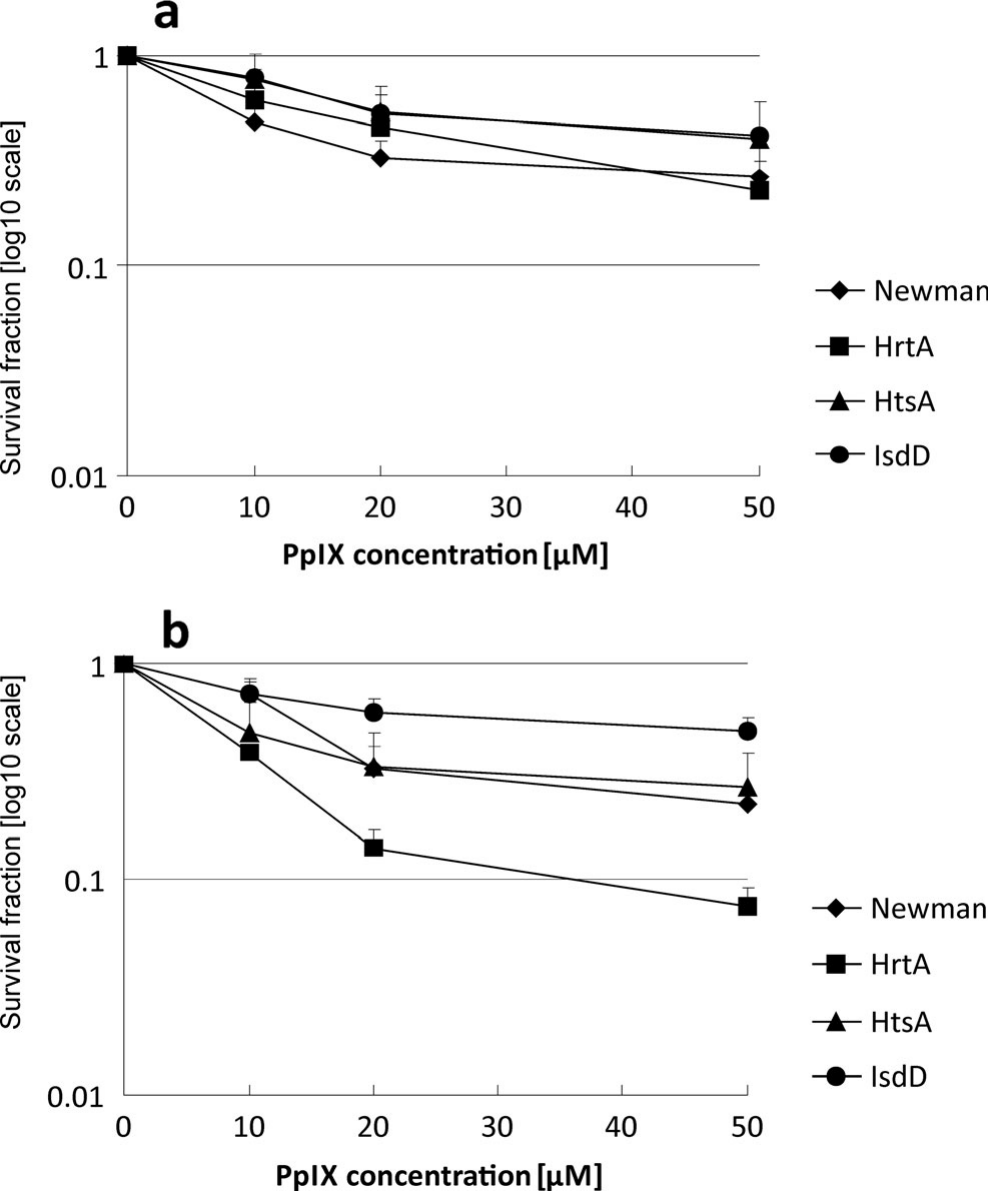
Survival of bacterial *S. aureus* cells after photodynamic treatment in the absence or presence of Fe^++^. Bacterial cells were cultured either in medium without iron ions (**a**) or containing iron ions (**b**). Cells were incubated with increasing concentrations of PpIX and illuminated with a 12 J/cm^2^ light dose (623 nm). Bacterial survival was measured by serially diluting cells and counting colony-forming units (CFUs) plated on agar plates before and after treatment. The survival fraction was expressed as the number of CFU obtained after PDI treatment with respect to the number of CFU of non-light-treated cells. The names of the *S. aureus* strains are indicated in the legend

In the first set of experiments, we applied the photodynamic inactivation of wild-type strain Newman and its three isogenic mutants, ΔHrtA, ΔHtsA and IsdD, to assess possible differences in vulnerability to PDI. We used PpIX, which is a structural analogue of haem and acts as a photosensitiser. We used monochromatic polarised red light (623 ± 23 nm, 98 % polarisation) and PpIX concentrations in the range of 0–50 μM. The total light dose used was 12 J/cm^2^. The reason for using small doses of light in this experiment was to maintain sublethal conditions to assure the viability of bacteria and thus the sufficient expression of the proteins of interest. The experiments were performed either in the presence or absence of iron ions; certain iron-haem transporters are produced in response to a limited amount of Fe^++^ (Mazmanian et al. 2003). The mortality of all of the studied strains was lower in the absence of Fe^++^ (Fig. 1). Reductions in survival at 0.38, 0.64, 0.39 and 0.57 log_10_ were observed for ΔisdD, ΔHrtA, ΔHtsA and the wild-type Newman strain, respectively. When Fe^++^ was present in the culture medium, the cells of the Newman strain, ΔHrtA and ΔHtsA were killed more efficiently (0.65, 1.13 and 0.57 log_10_ units of survival reduction, respectively), whereas in the case of ΔisdD, the cell viability remained unchanged. The largest difference in the rate of cell death with respect to the presence of Fe^++^ was observed for the ΔHrtA strain, where in the presence of iron ions, the viability of the cells was reduced twofold compared with cells in the non-Fe^++^ containing medium (0.64 vs. 1.13 log_10_ units of survival reduction). We also performed PpIX uptake studies with respect to the presence of Fe^++^ and did not observe a difference between these two conditions. In summary, ΔHrtA was the most PDI-susceptible strain in both the presence and absence of Fe^++^ (Fig. 1). Moreover, the observed effect was not related to strain-dependent differences in growth rate in the presence and absence of Fe^++^. The growth rate of each of the studied strains was reduced to the same extent in the absence of Fe^++^ (on the Supplementary material Fig. S2).

### Light-dependent PDI survival

**Fig. 2:**
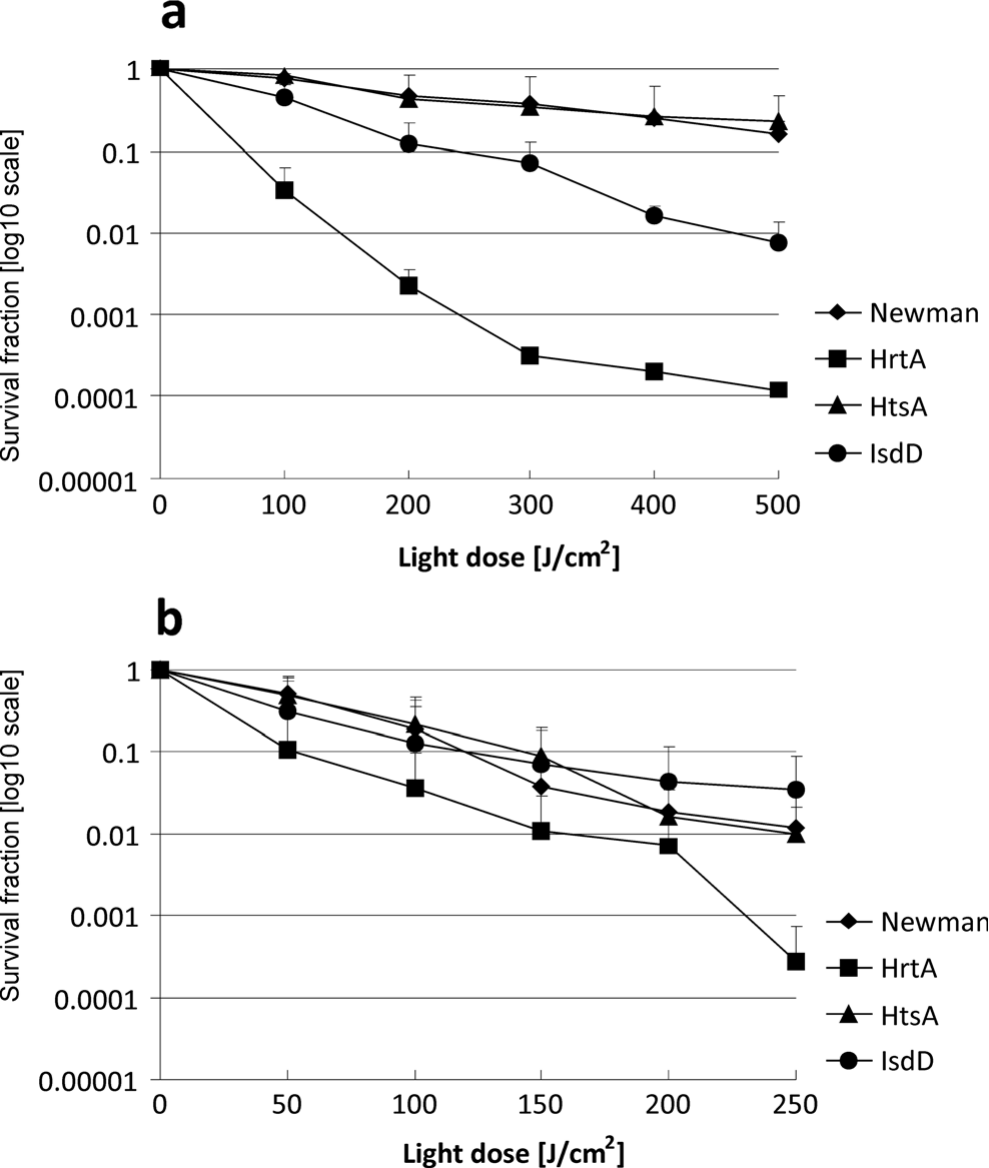
Light dose-dependent survival of bacterial *S. aureus* cells after photodynamic treatment. Survival of the wild-type Newman strain and its three isogenic mutants, ΔHrtA, ΔHtsA and ΔIsdD, were measured after PDI treatment with respect to increasing light doses. After incubation with 20 μM PpIX, the analysed strains were subjected either to 620–780 (**a**) or 385–480 nm (**b**) narrowband light. Bacterial survival of bacteria was measured by serially diluting cells and counting the CFUs plated on agar plates before and after treatment. The survival fraction is expressed as the number of CFU obtained after PDI treatment with respect to the number of CFU of non-light-treated cells. The names of the *S. aureus* strains are indicated in the legend

**Fig. 3:**
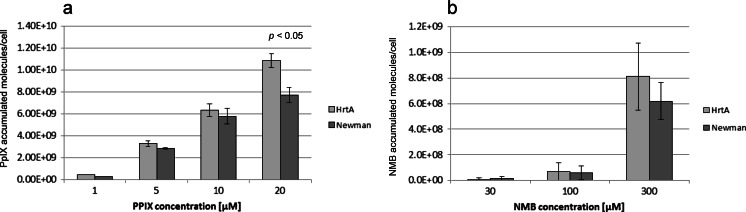
Protoporphyrin IX and new methylene blue accumulation. Uptake of two photosensitisers, namely PpIX (**a**) and new methylene blue (**b**), was studied using the reference *S. aureus* Newman strain and the *S. aureus* HrtA mutant. PS uptake was carried out in the presence of increasing concentrations, as indicated in the picture (PS concentration range of 1–20 μM for PpIX and 10–300 μM for NMB). Cells were incubated with PS at 20 °C for 30 min. The value of the accumulated PS is represented as the number of molecules per cell

**Fig. 4:**
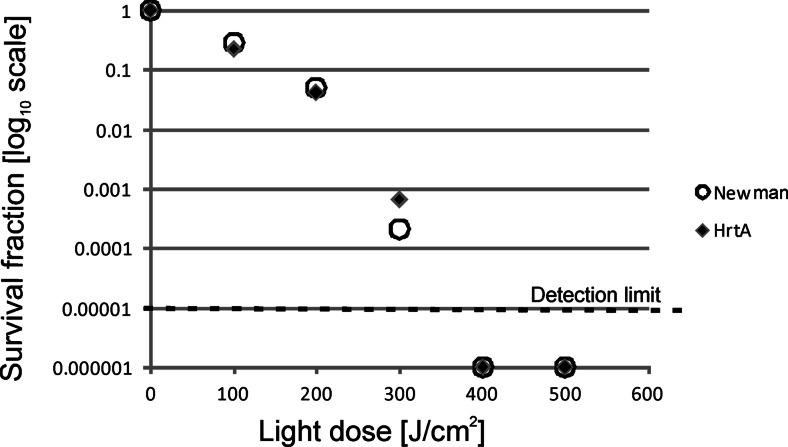
Light dose-dependent survival of bacterial *S. aureus* cells after photodynamic treatment. Survival of the wild-type *S. aureus* Newman strain and its isogenic *S. aureus* HrtA mutant strain were measured after PDI treatment with respect to increasing light doses. After incubation with 300 μM NMB, the analysed strains were subjected to 620–780 nm narrowband light. Bacterial survival was measured by serially diluting the cells and counting the CFUs plated on agar plates before and after treatment. The survival fraction is expressed as the number of CFU obtained after PDI treatment with respect to the number of CFU of non-light-treated cells. The names of particular strains are indicated in the legend

To characterise the photoinactivation process of the studied strains, we applied PDI survival assays with increasing light doses. Here, we used photoinactivation conditions, in which bacteriocidal effects could be obtained. We were interested if the applied lethal conditions similarly affect the analysed strains. We observed a light dose-dependent response to the treatment in the range of 0–500 J/cm^2^ for the red light and 0–250 J/cm^2^ for the blue light. Here, a halogen lamp was used with filters generating a narrow band of blue or red light (“Materials and methods” section). Both applied wavelengths refer to characteristic spectral properties of porphyrins, in which *Q* and Sorret bands of absorption spectra are distributed along the entire range of visible light. In each light dose-dependent experiment, we applied a 20 μM concentration of PpIX. Samples of the treated cells were taken at time intervals that corresponded to a particular light dose (Fig. 2). Again, the ΔHrtA clearly presented the most PDI-sensitive phenotype. The observed reduction in survival was 4 log_10_ units in viability, whereas in the wild-type Newman strain as well as in ΔHtsA strain, the observed reduction in survival was estimated to be less than 1 log_10_ unit. For ΔIsdD, the observed reduction in survival was 2 log_10_ units. When the blue light was applied, ΔHrtA again appeared to be the most susceptible strain to the treatment. This time, however, the differences in survival rates between the most vulnerable strain (ΔHrtA, 3.5 log_10_ units) and the remaining strains (1.5 log_10_ units for ΔIsdD and 2 log_10_ units for ΔHtsA and Newman) were not as significant as in the case of the red light treatment. We believe that it may be the result of the light penetration. The experiments with both types of light were performed in similar technical conditions, in 96-well plates. Red light penetrates deeper, and thus, the chances are bigger to reach also these bacterial cells that are located near the bottom of the flask. Next, we determined whether the decreased survival of ΔHrtA with respect to reference Newman strain correlated with the photosensitiser (PS) accumulation in mutant and wild-type cells. A significantly greater number of PpIX molecules were accumulated in ΔHrtA cells than Newman cells (Fig. 3a). Although the trend was observed within all the concentrations tested (1–20 μM PpIX), statistically significant difference was only found for the situation when cells were incubated with 20 μM PpIX (Fig. 3a). We also determined whether there were any differences in the accumulation of new methylene blue between the two strains. New methylene blue represents a heterocyclic aromatic chemical compound that differs from the tetrapyrrole structure of porphyrins. In this case, no difference in molecule accumulation was observed between the two strains studied (Fig. 3b). A detailed analysis of the survival of ΔHrtA cells and wild-type Newman cells after NMB-based photodynamic inactivation revealed no differences in the number of surviving cells (Fig. 4).

### ALA-induced endogenous photosensitiser production in cells

**Fig. 5:**
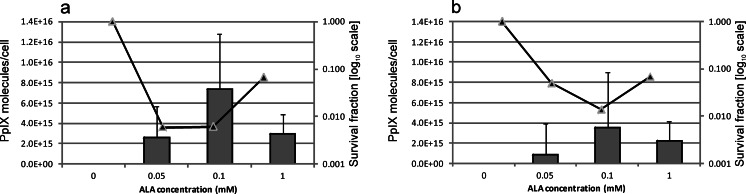
ALA-induced photodynamic inactivation and intracellular porphyrin content in *S. aureus. S. aureus* Newman (**a**) and its isogenic ΔHrtA mutant (**b**) were incubated for 4 h in the dark with 5-ALA. *Bars* represent the intracellular bacterial porphyrins (PpIX) produced and extracted from the cells. *Solid lines* (♦) represent the reduction in survival after photodynamic treatment with 50 J/cm^2^ blue light (385–480 nm). Survival fraction is expressed as the number of CFU obtained after PDI treatment with respect to the number of CFU of non-light-treated cells

Results of the photoinactivation and accumulation studies revealed that the ∆HrtA mutant had the most pronounced effect of photoinactivation when using exogenously added PpIX. To determine whether endogenously produced porphyrin-based photosensitisers impact PDI response in the studied wild-type Newman strain and the haem transporter mutant ∆HrtA, the cells were incubated with 5-ALA at concentrations of 0, 0.05, 0.1 and 1 mM. The 5-ALA compound is a precursor of porphyrin, which is known to cross the bacterial cell wall and induce endogenous porphyrin production (Kennedy and Pottier 1992). Endogenous porphyrins were extracted from the cells, and the fraction of surviving cells was estimated after illumination of 50 J/cm^2^ blue light. From these data, we observed a correlation between the amount of porphyrins produced in the cells and the extent of cell death after blue light treatment (Fig. 5). Interestingly, we observed that the most efficient 5-ALA concentration, i.e. the one that caused the highest porphyrin production, was 0.05 mM of the *S. aureus* Newman strain and 0.1 mM for the ∆HrtA strain. When higher concentrations were used, namely 1 mM, lower amounts of porphyrins were extracted from the cells. In every case, the 1-mM 5-ALA treatment resulted in reduced mortality after blue light treatment. Figure 5 shows that when endogenous photosensitisers are induced in *S. aureus* cells, the behaviour of the two analysed strains, namely wild-type Newman and the ΔHrtA mutant, is similar with respect to PDI susceptibility. Moreover, the extent of cell survival reduction correlated well with the amount of porphyrins induced after 5-ALA treatment. The observed reduction in survival of the analysed strains after 5-ALA treatment was in accordance with the results obtained from FACS analyses. Using this technique, the percentage of fluorescing bacterial cells after incubation with 5-ALA was estimated to be similar for these two strains (Table 1). This suggests that HrtA does not play a role in a 5-ALA-based PDI. It must be noted however that we have made rough estimation based on technical repeats. We did not perform statistical analysis on these results.

**Table 1:** Percentage of fluorescing cells after 5-ALA incubation obtained from FACS analysis

5-ALA concentration	0.01 mM	0.05 mM	0.1 mM	1 mM
*S. aureus* strain				
Newman	1.9 %	11.6 %	29.4 %	9.5 %
ΔHrtA	0.8 %	6.8 %	26.6 %	19.8 %

### Trypsin treatment affects PpIX uptake in ∆HrtA

**Fig. 6:**
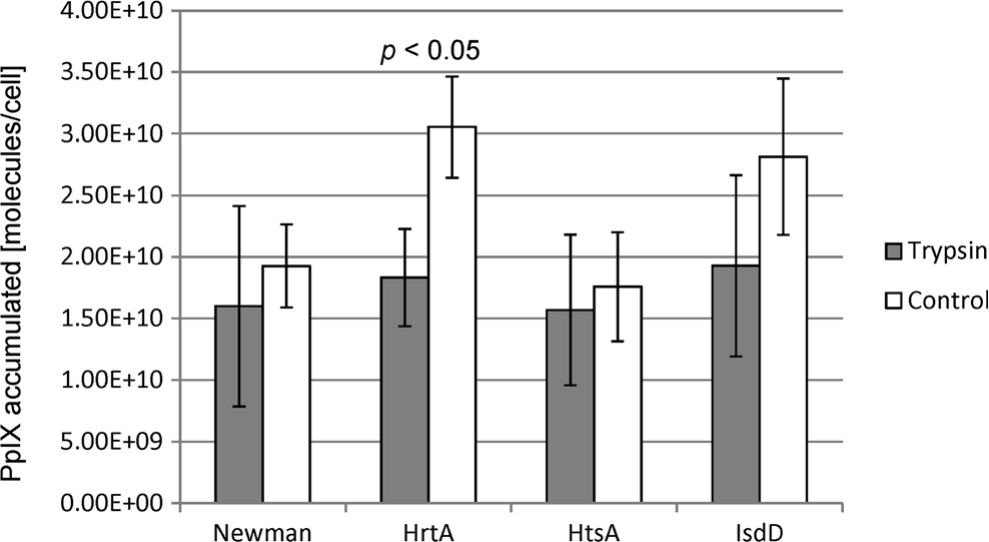
Trypsin treatment decreases protoporphyrin IX uptake. Uptake of PpIX was carried out in the presence of 10 μM of the photosensitiser. In the trypsin-treated cells (trypsin), the samples were incubated with 0.005 % (*w*/*v*) trypsin for 15 min, 37 °C; washed with PBS buffer; and further incubated with PpIX (30 min, 37 °C). In the non-treated samples (control), bacterial cells were incubated directly with PpIX without trypsin pre-treatment. *S. aureus* strains used in the experiments are listed in the legend

There is currently no specific inhibitor available for HrtA transporter; thus, we applied a trypsin treatment approach to analyse the participation of proteins in the process of photosensitizer uptake by bacterial cells. We incubated cells with 10 μM PpIX in the presence or absence of trypsin. The purpose of trypsin treatment was to ‘shave off’ cell wall-bound proteins to inactivate them and, therefore, prevent them from affecting PpIX uptake. The cells were incubated with trypsin for 15 min, followed by a 30-min incubation with the photosensitiser. Differences in PpIX uptake could be observed in the trypsin pre-treated cells compared with the non-trypsin-treated cells (Fig. 6). In all of the presented experiments, we employed *S. aureus* strain Newman and its three isogenic mutants that were defective in one of the haem transporter systems, namely HrtA, HtsA and IsdD. Although there was an overall trend of a higher PpIX uptake in the control cells (without trypsin addition) compared with the trypsin-treated cells, statistical significance was only found for ΔHrtA. The highest PpIX uptake value in the control cells was observed for ΔHrtA (3.05 × 10^10^ molecules/cell), followed by strain ΔIsdD (2.81 × 10^10^ molecules/cell) and the wild-type strain together with ΔHtsA (1.93 × 10^10^ and 1.76 × 10^10^ molecules/cell, respectively). In trypsin-treated strains, the lowest PpIX uptake values were observed for the ΔHtsA cells (1.57 × 10^10^ molecules/cell) and the wild-type Newman strain (1.6 × 10^10^ molecules/cell); slightly higher values were noted for ΔHrtA and ΔIsdD (1.83 × 10^10^ and 1.93 × 10^10^ molecules/cell, respectively). Overall, there were no significant differences in the accumulation of PpIX between the studied strains after trypsin treatment. To ensure that trypsin treatment did not affect the cellular conditions, the supernatants of trypsin-treated cells were analysed to investigate the possible differences in membrane permeability. We did not observed any differences in the treatment of a particular strain in our experimental conditions (on the Supplementary material Fig. S1).

### Cell membranes of Newman and ∆HrtA strains have various fluidities

**Fig. 7:**
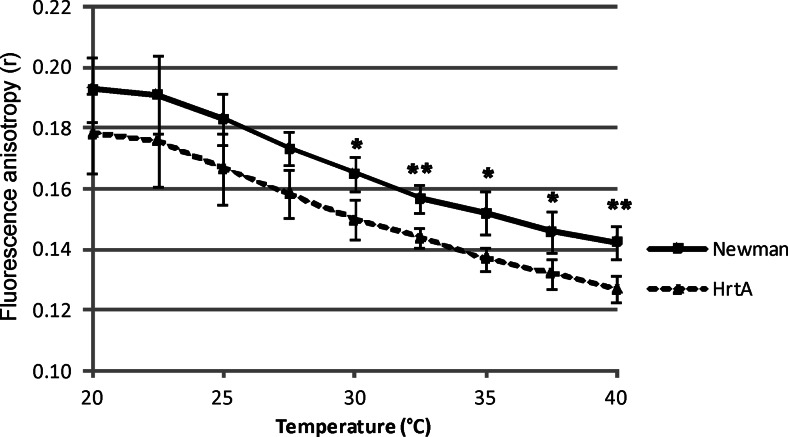
Cell membrane fluidity. The protoplasts of *S. aureus* Newman and its isogenic ∆HrtA mutant were incubated with the 1,6-diphenyl-1,3,5-hexatriene (DPH) probe, and fluorescence anisotropy (*r*) was measured, which inversely correlates with membrane fluidity. The values of fluorescence anisotropy were calculated based on fluorescence intensity measured with a vertical and perpendicular orientation of the analyser according to the equation described in the “Materials and methods” section. The names of particular strains are indicated in the legend. The presented values are the means of at least three biological replicates. *Statistically significant at the level of <0.05. **Statistically significant at the level of <0.01

The results of the photoinactivation experiments and the studies on PpIX accumulation did not explain the role of HrtA transporter in PDI. To explain the phenomenon of increased ∆HrtA vulnerability to the photoinactivation process when using PpIX, we assessed cell membrane fluidities in both strains. We employed a fluorescent probe to observe the fluidities of the cell membranes in the range of 20–40 °C. The DPH fluorescent probe was used, which provides a strong fluorescent signal in the hydrophobic core of the membrane but lacks fluorescence whilst in an aqueous environment. There is an inverse relationship between fluorescence polarisation values and cell membrane fluidity, which means that the lower polarisation indices, the greater the cell membrane fluidity (Bayer et al. 2000). As can be observed in Fig. 7, the strains differ with respect to their membrane fluidity (∆HrtA representing the more fluid state of the membrane compared to wild-type Newman). The observed difference was statistically significant at higher temperatures, i.e. >30 °C.

### Differing lipid content in Newman and HrtA strains

Bacterial membrane fluidity is associated with its lipid composition. Longer acyl chains, increased levels of unsaturation and shifts in iso- to anteiso-branched chain fatty acid content were shown to be associated with higher membrane fluidity (Klein et al. 1999). Following the observation that different fluidity is observed for the ∆HrtA mutant compared with a wild-type strain, we employed a mass spectrometry analysis to characterise the lipid profiles of the two analysed strains. We used the methodology described in our previous paper (Hewelt-Belka et al. 2014). We identified all of the main components of lipids present in cell membranes, namely phosphatidylglycerols (PGs), lysyl-phosphatidylglycerols (Lys-PGs), cardiolipins (CLs), diglycosyldiacylglycerols (DGDGs) and diacylglycerols (DGs). Identification was performed based on an automatic self-prepared database search and a manual investigation of acquired MS/MS spectra according to the previously described methodology (Hewelt-Belka et al. 2014). Interestingly, comparative lipidomics of two analysed strains revealed differences in lipid profiles of the two strains. The identified lipids, which differed significantly between the two strains, are shown in Table 2. We observed that DGs are present in the ∆HrtA strain at higher amounts compared with the Newman strain. However, the level of Lys-PGs is significantly higher in the wild-type Newman strain. An ambiguous pattern was observed for glycosyl diacylglycerols (DGDG) and monoglycosyldiacylglycerol (MGDG) (Table 2).

**Table 2:** Identification of lipid groups differing *S. aureus* Newman and ∆HrtA strains

Lipid group	Retention time (min)	Neutral mass	Ion	∆HrtA/Newman (fold change)	*p* test *U*
DG (15:0/15:0)	14.5596	562.4578	[M + Na]^+^	1.338038	0.008658
DG (15:0/17:0)	17.07775	590.4893	[M + Na]^+^	1.247986	0.008658
DG (15:0/19:0)	18.9791	618.5205	[M + Na]^+^	1.300394	0.008658
Lys-PG (15:0/18:0)	14.70278	864.6213	[M + H]^+^	0.618535	0.002165
Lys-PG (15:0/19:0)	15.91993	878.6369	[M + H]^+^	0.57844	0.002165
Lys-PG (15:0/20:0)	16.95452	892.6527	[M + H]^+^	0.622163	0.002165
DGDG (15:0/15:0)	10.55135	881.6081	[M + NH_4_]^+^	1.38726	0.002165
DGDG (15:0/17:0)	16.3492	909.6397	[M + NH_4_]^+^	1.289001	0.002165
DGDG (15:0/17:0)	11.43655	909.6376	[M + NH_4_]^+^	0.861046	0.015152
DGDG (15:0/18:0)	13.44875	923.6556	[M + NH_4_]^+^	0.792507	0.008658
DGDG (15:0/19:0)	14.42605	937.671	[M + NH_4_]^+^	1.186194	0.002165
DGDG (15:0/19:0)	14.84777	937.6709	[M + NH_4_]^+^	0.698586	0.008658
DGDG (15:0/20:0)	15.9671	951.687	[M + NH_4_]^+^	1.186194	0.004329
DGDG (17:0/20:0)	17.68063	979.7174	[M + NH_4_]^+^	0.718813	0.002165
MGDG (15:0/17:0)	14.21557	747.5866	[M + NH_4_]^+^	1.246444	0.002165
MGDG (15:0/18:0)	15.90033	761.6022	[M + NH_4_]^+^	0.817235	0.015152
MGDG (15:0/19:0)	16.59992	775.6177	[M + NH_4_]^+^	1.278867	0.008658
MGDG (15:0/20:0)	17.81935	789.6335	[M + NH_4_]^+^	0.834633	0.004329

*DGs* diacylglycerols, *Lys-PGs* lysyl-phosphatidylglycerols, *DGDGs* diglycosyldiacylglycerols, *MGDGs* monoglycosyldiacylglycerols

## Discussion

Iron uptake by bacteria is crucial for survival and pathogenicity. In humans, haem is the most abundant iron source. Bacteria use highly specific haem transport systems to capture it and transport it into the cell for utilisation. Bacteria have evolved several systems that enable efficient haem-iron transport. Some authors have proposed that protoporphyrin IX, which is structurally similar to a haem molecule, may be a substrate for the haem transport machinery (Moriwaki et al. 2011). In terms of photodynamic inactivation, this suggests that the response of bacteria to PDI may depend on specific proteins; thus, resistance may occur whilst treating the cells with a combination of sublethal doses of light and a photosensitiser. We hypothesised that haem transporters may participate in the process of photosensitiser accumulation to further influence the outcome of PDI.

The accumulation of a photosensitiser is a pre-requisite of photodynamic inactivation. The mechanism of porphyrin photosensitiser accumulation has primarily been studied with respect to eukaryotic cells, in which hydrophobic porphyrin photosensitisers mainly accumulated in the membrane fraction and caused severe damage following illumination compared with hydrophilic porphyrin photosensitisers (Sandberg and Romslo 1980). When we consider bacterial cells to be targets for photodynamic action, negatively charged porphyrins do not appear to be very effective because they easily aggregate in water solutions and are electrostatically repulsed by the negatively charged membrane. Here, we chose PpIX as a photosensitiser for our analysis because it is naturally produced by bacteria and it is the closest structural analogue of haem.

The decrease in PpIX accumulation after trypsin treatment was the highest for the ΔHrtA mutant, which may indicate a particular role of this phenotype in the photodynamic inactivation of *S. aureus*. We were interested in verifying our initial hypothesis that PpIX is a substrate for the HrtAB export system. Consequently, the inactivation of HrtA results in the accumulation of a higher amount of PpIX photosensitiser in the cells and thus in higher efficacy in PDI outcome. In the case of non-trypsin-treated ΔHrtA cells, the accumulation of the PS correlated well with the PDI response and the ΔHrtA strain was killed most efficiently in the PpIX-based PDI, both in a PpIX concentration-dependent manner as well as a light dose-dependent manner (Figs. 1 and 2). The lack of a specific inhibitor of the HrtAB transporting system precludes definite conclusions from these observations. Nevertheless, decreased accumulation of PpIX after trypsin treatment was only observed for the ∆HrtA mutant. The highest PPIX uptake was detected for the HrtA mutant strain. However, upon trypsin treatment, PpIX uptake was lower and similar for all the strains tested. If the function of HrtA is responsible for the observed PpIX uptake difference (trypsin treatment vs. no trypsin), then we would observe it in the wild-type Newman strain and not in the mutant, in which HrtA is innately absent. This indicates that the lack of a physical presence of the HrtA protein in the membrane (and not its function) is responsible for higher accumulation of PpIX, in addition to the higher efficacy of photoinactivation. Recent studies by Wakeman et al. (2014) showed that HrtAB was not capable of exporting toxic and non-toxic metalloporphyrins from *S. aureus* cells; however, PpIX itself was not studied. PS accumulation is known to lead to efficient PDI, but it is not always obvious that higher amounts of PS accumulation result in more effective PDI. For example, *S. aureus* and *Staphylococcus epidermidis* strains that produced biofilms were shown to accumulate more PS than their mutated (not producing biofilm) counterparts but were killed less efficiently with the use of chlorine(e6) as a PS (Gad et al. 2004). The charge and structure of the slime may induce PS trapping, thereby preventing it from reaching its effective destination (e.g. the membrane). This is likely not the case in our research, because all the studied strains are of the same genetic background and differ only according to the presence of particular haem transporters. However, based on the data from literature, the net charge and the structure of the bacterial cell surface may be disturbed when deleting membrane or cell surface proteins. This is exemplified by the observation that HrtB expression compromises the membrane structure in *S. aureus* strains that lack HrtA (Attia et al. 2010). The exposure of ΔHrtA to haem was demonstrated to affect the membrane structure but leave the cells viable.

There are several explanations for why ΔHrtA is the most susceptible strain to porphyrin-mediated PDI. One explanation may be related to the perturbations in a membrane, whereas others can be associated with an increase in protein content on the cell surface. When the ΔHrtA strain was challenged with haem, over 500 transcripts were shown to be upregulated, including several proteins that are associated with the cell wall, such as sortase A, fibronectin and fibrinogen-binding protein (more than 30-fold increase) (Stauff et al. 2008). Such proteins can represent an anchor for PpIX by trapping the photosensitiser near its site of action and further enabling efficient photokilling. However, the amount of PpIX accumulated by ΔHrtA after trypsin treatment was decreased compared with that in non-treated cells. In the remaining strains, including wild-type Newman, the PpIX accumulation level was unchanged (Fig. 6). Protoporphyrin IX was shown to interact with proteins such as human albumin and high-density lipoprotein (Kowalska et al. 2003). Porphyrin-mediated binding to haemoglobin and haemagglutinins was also demonstrated (Decarlo et al. 1999). However, in our experimental conditions, the roles of surface proteins in PpIX accumulation were minor. The second explanation for the higher susceptibility of the ∆HrtA mutant to PpIX may be associated with the properties of the bacterial membrane and a lack of HrtA protein. We observed differences in fluidity of the membrane of the two analysed strains. Additionally, the lipid content, which affects many cellular processes, varied between the two analysed strains. A lack of DGDG increased sensitivity to high light stress in *Synechocystis* (Mizusawa et al. 2009). and DGDG assists in protein-protein interactions in membranes (Domonkos et al. 2008). Potential differences in the glycosyl diacylglycerols (DGDG and MGDG) content in the analysed strains were ambiguous (Table 2). This precluded thorough interpretation of the results. However, the other two groups of lipids, namely DGs and Lys-PGs, showed a specific distribution pattern, the first one being overrepresented in the ∆HrtA mutant and the latter being more abundant in the wild-type Newman strain. The modification of the PG headgroup with lysine modulates the charge of the cytoplasmic membrane. PG lysinylation was shown to be correlated with reduced susceptibility of *S. aureus* to some antibiotics and cationic antimicrobial peptides due to increased positive charges of the membrane (Jones et al. 2008; Nishi et al. 2004; Peschel et al. 2001). Further detailed analysis on the model membrane system revealed a role of Lys-PG in the stabilisation of the membrane rather than the prevention of cationic antimicrobial binding (Kilelee et al. 2010). This is in accordance with our results in which the ∆HrtA mutant possessed significantly less Lys-PG and thus a less stable membrane than the Newman strain, which led to increased susceptibility to PDI and increased PpIX accumulation.

In the first part of our experimental work in which exogenous PpIX was used as a photosensitiser, we observed ΔHrtA to be the most vulnerable to PDI treatment. However, this was not the case when bacterial cells were incubated with 5-ALA, which induced the production of intracellular porphyrins (Nitzan and Kauffman 1999). The analysed transporter mutants expressed a pattern of PDI response very similar to that in the wild-type Newman strain when treated with 5-ALA (Fig. 5). Similar to previously published data (Lipovsky et al. 2009). we observed a relationship between the intracellular porphyrin content and the ALA-based PDI outcome in the strains presented in our paper. Notably, this phenomenon was not dependent on the presence or absence of the HrtA protein. Flow cytometry-based analyses conducted by us for the two analysed strains revealed that the percentage of fluorescing cells in each analysed strain was similar (Table 1), thereby confirming the previous data.

The most important conclusion based on the obtained results is that the haem transporter element, namely HrtA, is involved in the photodynamic inactivation of *S. aureus* cells with PpIX. The observed phenomenon is related to the high PpIX accumulation in *hrtA*-deleted cells, which further corresponds to an effective PDI outcome. We cannot exclude the possibility that PpIX is a substrate for the HrtAB efflux pump, which exports membrane-localised PpIX and therefore plays an important role in the *S. aureus* response to PpIX-based photodynamic inactivation. However, we also observed that a lack of the HrtA subunit alters bacterial cell membrane composition and fluidity, which sensitises *S. aureus* to photodynamic treatment. Our results highlight the role of the physical properties of the membrane as a molecular target for PpIX in photodynamic inactivation. It will be of interest to determine whether the physical properties of the membrane are also important for interactions between other photosensitisers and the cellular membrane.

## Electronic supplementary material

ESM 1 (PDF 565 kb)
