# Supplementary material for: Photoinactivation of Staphylococcus aureus using protoporphyrin IX: the role of haem-regulated transporter HrtA
Source: Appl Microbiol Biotechnol. 2015 Dec 3;100:1393–405. doi: 10.1007/s00253-015-7145-5 (PMC4717162; doi:10.1007/s00253-015-7145-5)
Supplement: Supplementary file 1 — (PDF 565 kb) [file 253_2015_7145_MOESM1_ESM.pdf]

Applied Microbiology and Biotechnology

Photoinactivation of *Staphylococcus aureus* using protoporphyrin IX: the role of haem-regulated transporter HrtA.

Joanna Nakonieczna*, Monika Kossakowska-Zwierucho, Michalina Filipiak, Weronika Hewelt-Belka, Mariusz Grinholc, Krzysztof Piotr Bielawski

[1]Intercollegiate Faculty of Biotechnology, University of Gdansk and Medical University of Gdansk, Kladki 24, 80-822 Gdansk, Poland

[2] Faculty of Biology, University of Gdansk, Wita Stwosza 59, 80-308, Gdansk, Poland (current address)

[3]Faculty of Chemistry, Gdansk University of Technology, Gabriela Narutowicza 11/12, Gdansk, Poland

* Corresponding Author: Joanna Nakonieczna

Telephone: +48 58 5236332

Fax: +48 58 5236426

Email: joanna.nakonieczna@biotech.ug.edu.pl

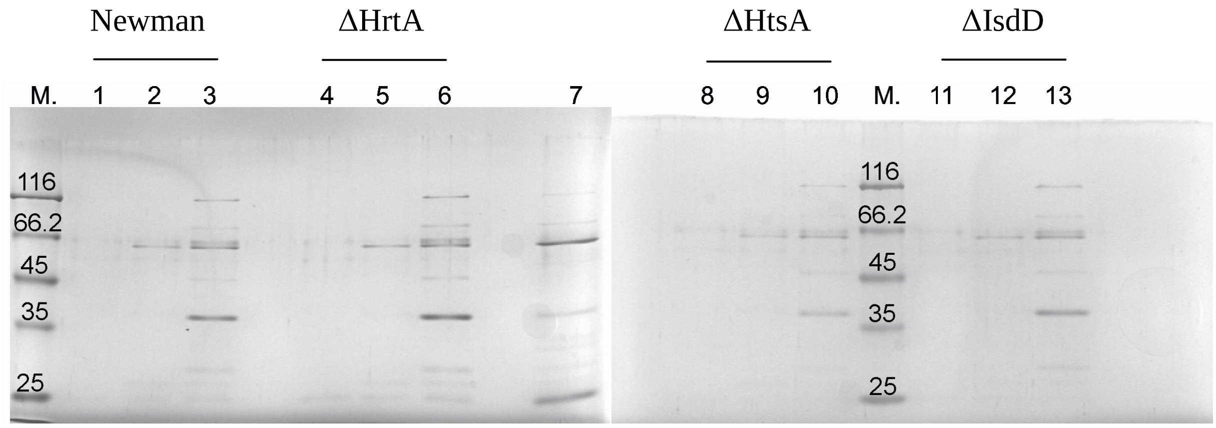

Fig. S1 Membrane integrity after trypsin treatment. In the experiments with trypsin treatment, cells were grown overnight and adjusted to OD_{600} = 0.3, 800 µl bacterial aliquots were centrifuged (1 min, 7500xg), and further dissolved in the same volume of 0.005% (w/v) concentration of the trypsin (dissolved in H_2O or PBS). Following incubation with trypsin (15 min, 37˚C), cells were centrifuged (2 min, 7500xg), and 20 µl of the supernatant was loaded on the gel. Four strains were analyzed, namely: *Staphylococcus aureus* Newman, ΔHrtA, ΔHtsA, ΔIsdD as indicated in the picture. Lanes: 1, 4, 8, 11 represent cells treated with trypsin dissolved in water, lanes: 2, 5, 9, 12 represent cells treated with trypsin dissolved in PBS, lanes: 3, 6, 10, 13 represent cells treated with PBS without trypsin. Lanes: M. represent protein molecular weight markers of 116 kDa, 66.2 kDa, 45 kDa, 35 kDa, 25 kDa, respectively.

As can be seen in Lanes 3, 6, 10, 13 some proteins are visible in supernatants of intact cells, incubated in PBS without trypsin. This may result from the fact that stationary phase cells (overnight cultures) were used for the experiment, therefore some proteins maybe observed as a result of natural cell envelop damage.

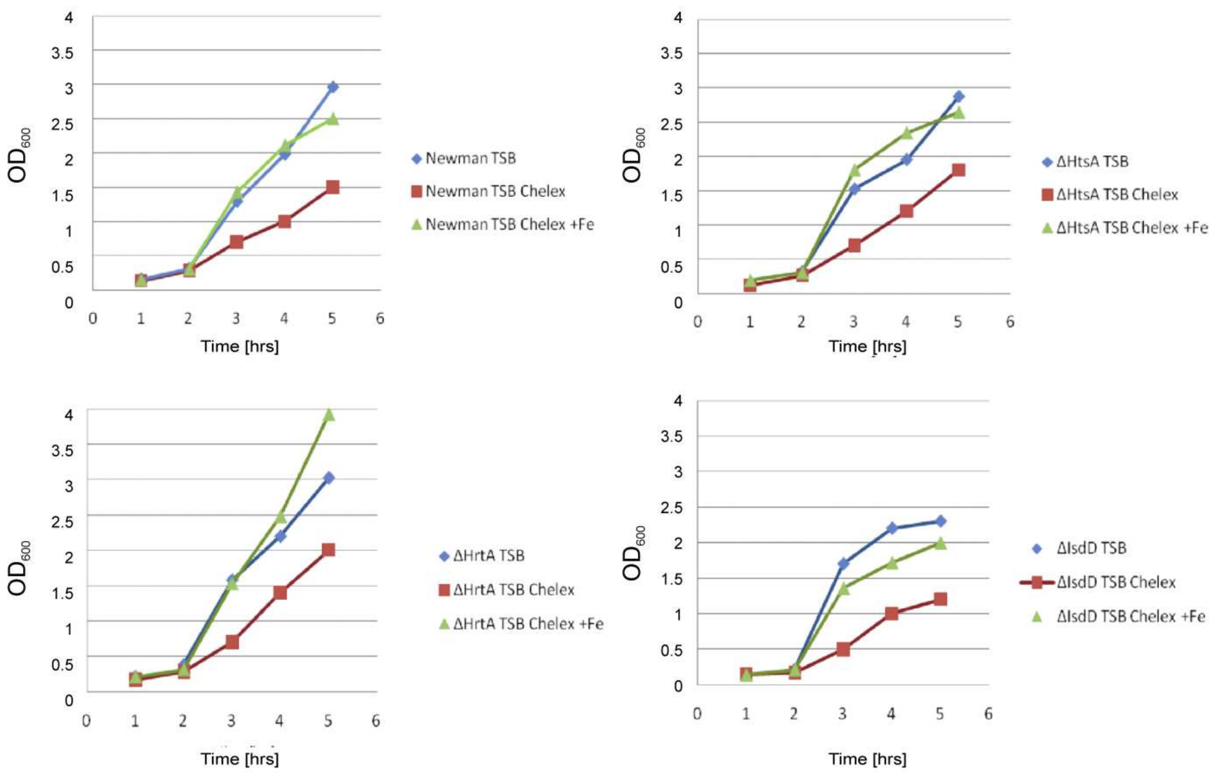

Fig. S2 Growth curve analyses of *Staphylococcus aureus* Newman, ΔHrtA, ΔHtsA, and ΔIsdD cultures in the presence or absence of Fe^{++}. Bacterial strains were cultured overnight in 5 ml TSB. Cells were diluted with fresh medium in a ratio 1:30, and cultured for next 6 hours in 37 ˚C, 200 rpm. Cultures of each strain analysed were carried out in TSB medium (**TSB**), or TSB medium treated with Chelex™ (**TSB Chelex**), or TSB medium treated with Chelex™ and further supplemented with $FeSO_4$ (**TSB Chelex + Fe**). The growth rate of each strain was decreased in TSB medium without Fe^{++} ions, and was restored after Fe^{++} supplementation.
